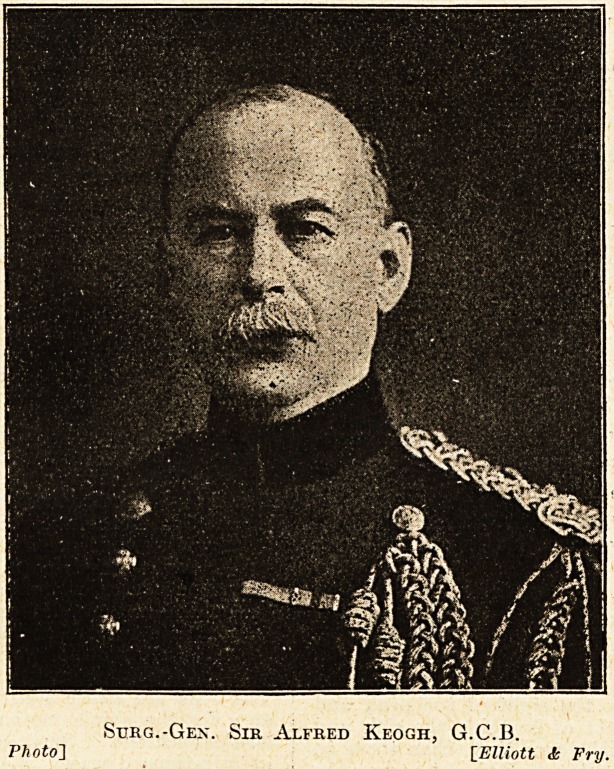# Sir Alfred Keogh, G.C.B., M.D.: The Director-General of the Army Medical Service

**Published:** 1917-02-10

**Authors:** 


### SIR ALFRED KEOGH, G.C.B., M.D.

### The Director-General of the Army Medical Service.

The bestowal a fortnight or so ago upon Sir Alfred Keogh, the Director-General of the Army Medical Service, of the Grand Cross of the Order of the Bath has not only been received throughout the Empire with an enthusiasm rarely evoked by the decoration of any but victorious generals, but also produced a few days later a tribute from Lord Esher which contains certainly one of the most remarkable avowals that have ever fallen from British administrators and publicists. As Lord Esher more than once remarks in his letter published in the *Times* of February 3, the time has not yet arrived when the debt which the nation owes to Sir Alfred Keogh can be fully disclosed or adequately acknowledged. Much that is at present gossip and guesswork will some day be public property, and it would be unwise and improper to attempt to anticipate that day. In what follows, therefore, it will be understood that the full tale of his work and accomplishments is understated rather than the reverse.

Surg.-Gen. Sir Alfred Keogh, G.C.B.
*Photo*] [*Elliott & Fry.*

### Creative Activities.

When Surgeon-General Keogh was placed at the head of the Army Medical Service in 1904 he was promoted over the heads of a good many senior officers; he was, in fact, but forty-six years of age. The War Office and the whole organisation of the British Army were to some extent in the melting-pot, as the lessons of the South African War were being digested and turned to account. Thus the new Director-General had a fine chance of proving his mettle, and right well the (comparatively) young Surgeon-General used it. It is not too much to say that he shaped the Royal Army Medical Corps and the Army Medical Service into their present form, and that in many respects he was far more prescient than those under whom he had to work. This was the more remarkable in that the South African War, if we remember rightly, was his first experience of active service, and that his administrative work there was confined to the command of a base hospital; for his services during this campaign he became a Companion of the exalted Order in which he is now advanced to the First Class.

Lord Esher's letter, to which we shall presently refer, bears testimony to the correctness of the principles upon which Sir Alfred's constructive work at the War Office was based. One small anecdote may be quoted as a further example of the same point. Whether the story is strictly accurate or not, at any rate it is believed in the R.A.M.C., and was told in good faith to the writer by a senior officer of that corps. The story goes that Sir Alfred was instructed, when he was drawing up the scheme of medical organisation of the old Expeditionary Force, to provide for a certain percentage of casualties per Division. He represented to the authorities that the casualties likely to arise should the Force ever be engaged in a European war would be at least twice the number he was asked to provide for, but was told to mind his own business, or words to that effect, and budget for casualties as instructed. The story goes on that Sir Alfred was so sure of his own judgment that he deliberately planned his field ambulances and other arrangements to deal with the casualties which he himself expected, though for appearances he had to represent them as designed for half that number. If this story is true, Sir Alfred Keogh must be credited with foreseeing ten years beforehand the nature of the fighting in the present European war more accurately than the military chiefs of the War Office.

### Sir A. Keogh's Recall.

Sir Alfred's term as Director-General was extended beyond the usual prescribed duration of that office; and even when it terminated he was still comparatively a young man. It is no secret that neither of his two successors in the appointment possessed anything like his genius, energy, or capacity for adminstration and affairs. When war broke out he had been for more than four years on the retired list, and had been, in fact, holding an administrative appointment at the Imperial College of Science and Technology. As Lord Esher states, within a month or so after the outbreak of war it was realised that Keogh's recall was imperative; none but Odysseus, it was found, could bend the bow which he himself had fashioned. It did not require the testimony of a distinguished public servant to establish that fact; the mere appointment was in itself sufficient proof of what Lord Kitchener and the War Office authorities felt about the Director-Generalship. Lord Esher, however, goes further than that, in words which constitute one of the finest testimonials any medical officer has ever received.

Lord Esher's Avowal.

Lord Esher (who is now Sub-Commissioner of the British Red Cross in France) states that when his Committee was reconstituting the War Office Sir Alfred Keogh visited him and protested " in warm and almost impassioned language " against the proposal to place the Director-General of the Army Medical Service under the Adjutant-General. The protest was in vain, and the Army Medical Service is still under the control of the Adjutant-General. Lord Esher now acknowledges that Sir Alfred was right and he was wrong. With a courage and manliness which command high respect he says, " How much of the suffering undergone by our soldiers then " (1914) " and since was due to the short-sightedness of my Committee, and notably of myself, will never be known. Certainly the control of the Adjutant-General's branch of the R.A.M.C. was and is responsible not only for the early failure to grip the medical factors of this war, but for the hampering conditions under which Sir Alfred Keogh has worked. His triumphs, and those of the R.A.M.C., have been achieved in spite of obstacles that the subordination of science to ignorance, of elasticity to military discipline, explains but cannot justify."

Lord Esher ends this notable letter with an appeal to Lord Derby to strengthen the Army Council by placing the D.G.M.S. upon it. " Thus," he concludes, " would the work of Sir Alfred Keogh be happily recognised, to the infinite advantage of our sick and wounded to-day and to-morrow." Whether this suggestion will prove agreeable to those in authority it would be futile to guess; but the terms in which it is drawn up are such as no one can read without conceiving even higher respect for Sir Alfred Keogh, G.C.B., if possible, than that in which he has hitherto been held. As head of the gigantic organisation for caring for the sick and wounded of the British Army in this war, he stands far beyond all competition as the super-man of the British hospital world. No other Englishman has ever borne such a burden of responsibility in connection with British hospitals; and it is to be hoped that when once this war is over, none will ever be in a position to challenge his pre-eminence in this respect in the years to come.

## Figures and Tables

**Figure f1:**